# IL-1β, IL-6, and RANTES as Biomarkers of Chikungunya Severity

**DOI:** 10.1371/journal.pone.0004261

**Published:** 2009-01-21

**Authors:** Lisa F. P. Ng, Angela Chow, Yong-Jiang Sun, Dyan J. C. Kwek, Poh-Lian Lim, Frederico Dimatatac, Lee-Ching Ng, Eng-Eong Ooi, Khar-Heng Choo, Zhisheng Her, Philippe Kourilsky, Yee-Sin Leo

**Affiliations:** 1 Singapore Immunology Network, Agency for Science, Technology and Research (A*STAR), Singapore, Singapore; 2 Tan Tock Seng Hospital, Communicable Disease Centre, Singapore, Singapore; 3 Environmental Health Institute, Singapore, Singapore; 4 DSO National Laboratories, Singapore, Singapore; 5 Institute for Infocomm Research, A*STAR, Singapore, Singapore; New York University School of Medicine, United States of America

## Abstract

**Background:**

Little is known about the immunopathogenesis of Chikungunya virus. Circulating levels of immune mediators and growth factors were analyzed from patients infected during the first Singaporean Chikungunya fever outbreak in early 2008 to establish biomarkers associated with infection and/or disease severity.

**Methods and Findings:**

Adult patients with laboratory-confirmed Chikungunya fever infection, who were referred to the Communicable Disease Centre/Tan Tock Seng Hospital during the period from January to February 2008, were included in this retrospective study. Plasma fractions were analyzed using a multiplex-microbead immunoassay. Among the patients, the most common clinical features were fever (100%), arthralgia (90%), rash (50%) and conjunctivitis (40%). Profiles of 30 cytokines, chemokines, and growth factors were able to discriminate the clinical forms of Chikungunya from healthy controls, with patients classified as non-severe and severe disease. Levels of 8 plasma cytokines and 4 growth factors were significantly elevated. Statistical analysis showed that an increase in IL-1β, IL-6 and a decrease in RANTES were associated with disease severity.

**Conclusions:**

This is the first comprehensive report on the production of cytokines, chemokines, and growth factors during acute Chikungunya virus infection. Using these biomarkers, we were able to distinguish between mild disease and more severe forms of Chikungunya fever, thus enabling the identification of patients with poor prognosis and monitoring of the disease.

## Introduction

In the recent years, emerging and re-emerging tropical infectious diseases have been shown to cause high social and economic impact. Vector-borne infectious diseases such as Dengue, West Nile have been resurging largely due to the spread of insecticide resistance, to socio-demographic changes, and to genetic mutations in the pathogens. More recently, chikungungya fever (CHIKF) has now emerged as the next important infection in South-East Asia, the Pacific region and Europe [1]–[5], making it a major threat that requires immediate attention. Recent epidemic resurgence of CHIKF in several African and Asian countries demonstrated that infection can spread alarmingly rapidly [6]–[9] from limited early transmission that then developed into an unprecedented and unexpected epidemic, infecting 38% of the population as occurred in Reunion island [6], [8]. The appearance of cases in Europe, the United States and other countries by travelers returning from known outbreak areas underscores the contributory factors of increased human mobility, tourism, global climate change, and increases in insecticide resistance [10]–[15]. In this era of globalization, the threat of such disease epidemics should not be underestimated as such public health events could cripple public health systems and economies.

Chikungunya virus (CHIKV), which causes CHIKF, is an alphavirus of the *Togaviridae* family, with a 12,000-nucleotides linear, positive-sense, single-stranded RNA genome containing two large open reading frames (ORF). The first, ORF1, encodes 4 non-structural proteins (nsP1, nsP2, nsP3 and nsP4) while ORF2 encodes structural proteins that include 1 capsid protein (C), 2 major envelope surface glycoproteins (E1, E2) and 2 small proteins (E3, 6K) [8], [9]. CHIKV is transmitted by *Aedes* mosquitoes (mainly *A. albopictus* and *A. aegypti*).

CHIKF is an acute illness with abrupt fever, skin rash, arthralgia, and occasional involvement of the nervous system, heart and liver. Prolonged incapacitating arthralgia has sometimes been reported to persist for years [8], [9], [13]. It is of concern that the re-emerged CHIKV has caused considerable morbidity and some fatalities, whereas previously CHIKF was considered as relatively benign. Despite the fact that the clinical features of recent acute CHIKV infections from several countries have been described [16]–[18], little is known about the long-term sequelae or the pathogenesis of arthropathy, and the acquisition of protective immunity remains unexplored. It has been proposed that CHIKV-induced arthritis or arthralgia is of immunopathologic origin [19], [20]. At present, there is no specific or effective treatment for CHIKF, and patient management is largely symptomatic relief and primarily anti-inflammatory drugs [8]. Given the expanding geographic range of CHIKV and its potential to rapidly cause large scale epidemics, it has become important to understand the immune and pathogenic mechanisms active during CHIKV infections in order to guide the development of targeted and effective control and treatment strategies.

Cytokines and chemokines are thought to play an important role in viral immunopathology. Although IL-2. IL-10 and IFN-γ have been implicated in the pathogenesis of CHIKF [21], a global analysis of their specific involvement with disease severity has not yet been defined. Growth factors are usually produced in response to injury. Viral infections such as CHIKV, induce cellular damage which may lead to secretion of these factors; however, limited studies have been conducted [21]. In this study, we took the opportunity to conduct a detailed study on the patients from the first outbreak of CHIKF in Singapore [22], [23]. We measured circulating levels of a wide range of cytokines, chemokines, and growth factors in 10 laboratory confirmed cases of CHIKF, and compared them with healthy individuals. We next determined which biomarker was associated with infection and/or severity. We showed for the first time that CHIKV infection induced a wide range of cytokines, chemokines, and growth factors. We subsequently found that 3 specific biomarkers, namely IL-1β, IL-6, and RANTES levels, were associated with severe CHIKF.

## Results

All 10 patients included in this study were males. Their age ranged from 22 to 65 years (median, 35 years). All except one were foreign nationals. Half of our patients were classified as severe CHIKF. We defined severe CHIKF as having a temperature of >38.5°C or pulse rate >100/min, or platelet count <100×10^9^ g/L based on studies defining severe diseases [24]–[28]. Except for the Singapore resident, none had any pre-existing medical condition. Despite being previously healthy, four non-residents developed severe illness. Fever lasted 2–10 days, and fever duration was not significantly different between those who had more severe illness and those who had not (mean, 6.6 days vs. mean, 3.8 days; *P*>0.05). Two patients reported persistent arthralgia lasting more than two weeks. Demographic and clinical details of the 10 CHIKF patients are summarized in Table 1.

**Table 1:** Demographic and epidemiologic data on 10 patients with PCR-confirmed chikungunya infection.

Patient No.	Age (years)	Gender	Nationality	Duration of fever (days)	Illness severity^a^	Pre-morbid condition	Clinical Outcome^b^
1	45	M	Bangladeshi	2	Not Severe	None	Persistent arthralgia
2	32	M	Bangladeshi	4	Not Severe	None	Complete recovery
3	33	M	Indian	5	Not Severe	None	Complete recovery
4	45	M	Indian	5	Not Severe	None	Complete recovery
5	28	M	Malaysian	3	Not Severe	None	Complete recovery
6	65	M	Singapore resident	10	Severe	Liver cirrhosis, hypertension, atrial fibrillation, anaemia	Complete recovery
7	34	M	Indian	4	Severe	None	Complete recovery
8	39	M	Indian	6	Severe	None	Persistent arthralgia
9	37	M	Indian	9	Severe	None	Complete recovery
10	22	M	Malaysian	4	Severe	None	Developed myalgia

a Severity was defined as having a temperature >38.5°C or pulse rate >100/min or platelet count <100×10∧9/L.

b Clinical outcome at 2 weeks post-illness onset.

Among our patients, the most common clinical features were fever (100%), arthralgia (90%), rash (50%), and conjunctivitis (40%) (Table 2). Gastrointestinal and constitutional symptoms were less prominent. Arthritis was observed in only one patient, who had an effusion on the right knee. None had neurologic involvement or hemorrhagic manifestation.

**Table 2:** Clinical features.

Sign/Symptom	No. (%) of patients
Fever	10 (100)
Arthralgia	9 (90)
Rash	5 (50)
Conjunctivitis	4 (40)
Gastrointestinal symptom^a^	3 (30)
Headache	3 (30)
Eye pain	2 (20)
Back pain	2 (20)
Mylagia	1 (10)
Arthritis	1 (10)

a Nausea, vomiting, diarrhoea, or abdominal pain.

Table 3 presents a summary of the key laboratory findings among our patients throughout the course of their illness. White cell count, hemoglobin, hematocrit, platelet count, erythrocyte sedimentation rate for most patients were within the normal range. The mean nadir platelet count (±SD) was 199±115×10^9^/L. Only one patient had severe thrombocytopenia (nadir platelet count, <100×10^9^/L) during the course of his illness. Elevated C-reactive protein levels (CRP, >10.0 mg/L) were observed in 60% of patients, but the peak C-reactive protein level was not significantly different between those who classified as severely ill and those who were not (mean, 40.3 mg/L vs. mean, 9.9 mg/L; *P* = 0.195). The mean peak alanine and aspartate transaminases (ALT and AST) (±SD) were 58±36 U/L and 50±25 U/L respectively. Both ALT and AST were 2-fold greater than the upper limit of normal in one patient, who had pre-existing liver cirrhosis. None of the patients had a clinically abnormal total protein, urea or creatinine level. Among our patients, the mean nadir protein level (±SD) was 67±5 g/dL, and the mean peak urea and creatinine levels (±SD) were 5.1±1.7 mmol/L and 101±16 mmol/L respectively. Lactate dehydrogenase level was the only laboratory parameter that was significantly higher in severely ill patients, compared to those who were not (mean, 732 U/L vs. mean, 525 U/L; *P = 0.047*).

**Table 3:** Laboratory parameters in chikungunya confirmed patients.

Variable	Normal range	Mean±SD
Nadir white cell count, ×10∧9/L	3.6–9.3	4.5±1.1
Nadir hemoglobin, g/dL	13.0–17.0	14.6±1.3
Peak hematocrit, %	41.0–51.0	46.6±3.4
Nadir platelet count, ×10∧9/L	170–420	199±115
Peak erythrocyte sedimentation rate (ESR), mm/hr	1–10	7±8
Peak C-reactive protein (CRP), mg/L	0.0–5.0	26.8±33.7
Peak alanine transaminase (ALT), U/L	17–63	58±36
Peak aspartate transaminase (AST), U/L	15–41	50±25
Nadir total protein, g/dL	63–81	67±5
Peak urea, mmol/L	2.9–9.3	5.1±1.7
Peak creatinine, umol/L	60–110	101±16
Peak lactate dehydrogenase (LDH), U/L	250–580	654±50

**Figure 1:**
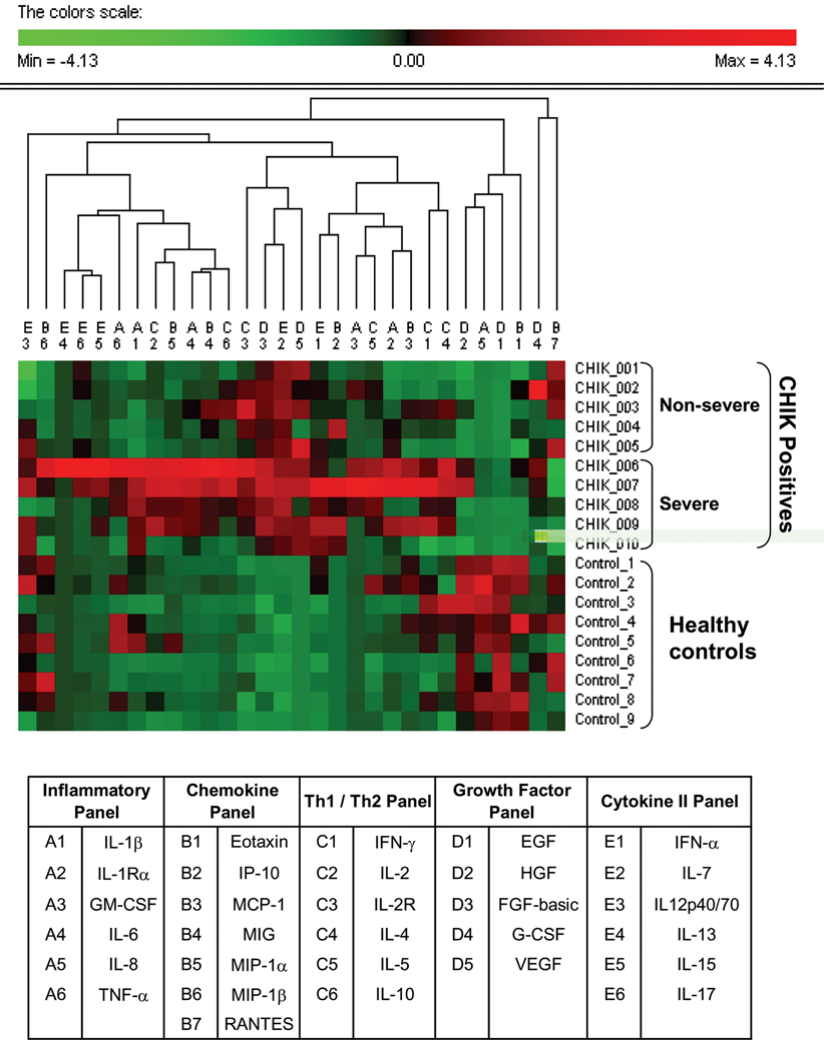
Two-way hierarchical clustering analysis. Each cell in the 2-dimensional graph indicates the measure of a single mediator in 1 sample, with standardized levels indicated by color according to the scale on the top. Sample clustering resulting from the algorithm described is shown at the right side of the graph, with an indication of the group to which each individual sample belongs. Mediator clustering is depicted on the top of the graph, and detailed at the bottom.

**Figure 2:**
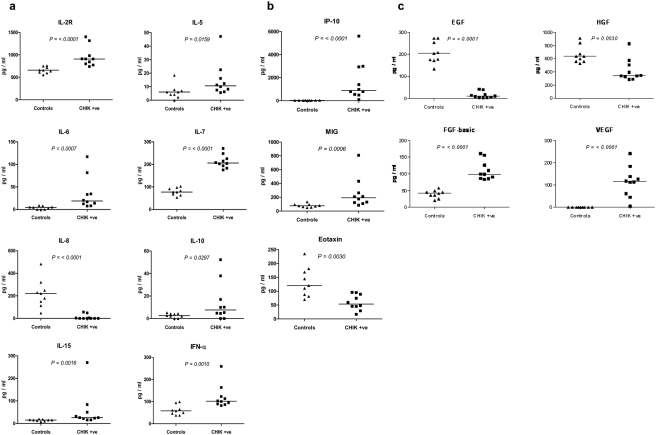
Differences in plasma mediator levels in CHIKF patients and healthy controls. a. Levels of cytokines (pg/ml) were determined as described and only those with a *P* value of *<0.05* are illustrated. Horizontal bars indicate the respective groupwise medians. b. Levels of chemokines (pg/ml) were determined. c. Levels of growth factors (pg/ml) were determined.

Profiles of 30 cytokines, chemokines and growth factors were determined by a multiplex-microbead immunoassay on acute blood samples collected upon hospitalization. The samples collected ranged from day 2 to day 19 of illness (median, day 4.5). To characterize the overall patterns, a two-way hierarchical clustering analysis was done to allow the classification of individuals according to disease severity based on the clinical features (Figure 1). Evidently, this had the power to discriminate the clinical forms of CHIKF in the samples in this study from the healthy controls, with patients classified as non-severe and severe disease segregating perfectly. The levels of 8 plasma cytokines (IL-2R, IL-5, IL-6, IL-7, IL-8, IL-10, IL-15 and IFN-α) were observed to be most significantly elevated (Figure 2a) in CHIKF patients compared to uninfected subjects (*P<0.05*). Among these was proinflammatory cytokine, IL-6 which was very significant. Interestingly, another proinflammatory cytokine, IL-8 was down-regulated in these patients. Anti-inflammatory cytokine, IL-10 was found to be significantly raised in most of the patients (*P<0.05*). The plasma concentrations of IL-2R and IL-5 were found to be increased in all patients. Levels of IFNα and IL-7 were elevated in all patients. The levels of other cytokines such as IL-2, IL-4, IL-12, IL-13, IL-17, IFN-γ, and TNF-α, were only marginally increased in the CHIKF group compared with those in the uninfected group (Figure S1).

Profiles of chemokines, IP-10 and MIG were shown to be significantly elevated, while Eotaxin was suppressed (Figure 2b). There was no difference in the levels of other chemokines namely, MCP-1, MIP-1α, MIP-1β and RANTES (Figure S1).

Interestingly, the levels of 4 growth factors were found to be significant in the patients, with up-regulation of HGF, FGF-basic and VEGF, with the exception of EGF which was almost totally suppressed (Figure 2c). It was observed that the CHIKF patients exhibited low levels of GM-CSF and G-CSF (Figure S1).

**Figure 3:**
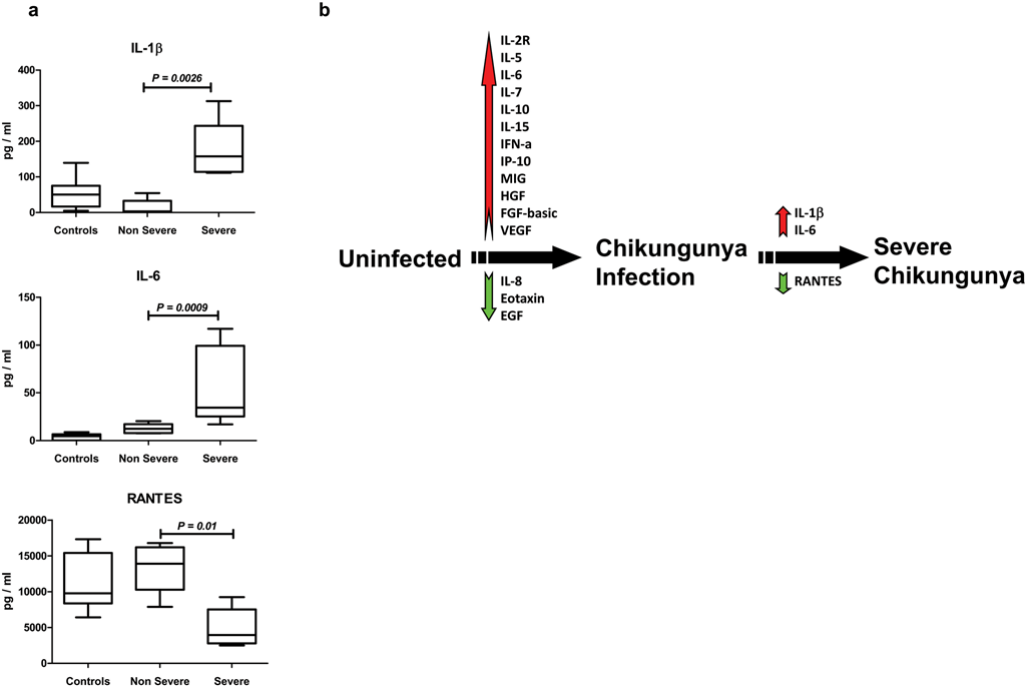
Differences in cytokines, chemokines and growth factors levels determine disease severity. a. Box-and-whisker plots illustrating the significant differences of IL-1β, IL-6 and RANTES in patients with non-severe and severe CHIKF. b. Diagrammatic representation of the mediator profiles in CHIKV-infected patients and healthy control subjects, and the distinction between non-severe and severe CHIKF.

Finally, in an effort to identify cytokine, chemokine, and growth factor plasmatic levels associated with severity, statistical analyses were performed after stratification of the CHIKF patients according to severity. It was observed that an increase in levels of IL-1β and IL-6, and a decrease in RANTES respectively were associated with disease severity (Figure 3a). The levels of all other markers were not significantly different (Figure S2 and S3).

## Discussion

CHIKF, an emerging arboviral infection, which induces high fever, has only been recently reported in Singapore. Up to Dec 2007, all CHIKF patients had contracted the infection overseas [22]. The first local outbreak of CHIKF occurred in Jan 2008. More than 2,500 people who lived or worked in the outbreak area were screened and a total of 13 PCR-confirmed cases were identified [22], [24]. All confirmed CHIKF cases were referred to the CDC/TTSH. Our report included 10 patients who participated in this study.

Phylogenetic analysis of the viral sequences of our patients has revealed that the circulating strains were of the Indian Ocean genotype and closely related to those from the 2006 outbreak in India [29] but without the A226V mutation, further emphasizing how remarkably rapid the disease could spread with the right environmental conditions. The attack rate in our outbreak was 0.5%, much lower than the 34% reported in Reunion Island and the 5.4% observed in Italy [13], [30]. This could be attributed to the rapid removal of human reservoirs through isolation, enhanced vector control, or the circulation of a virus strain of lower epidemic potential. Clinical features of our patients were similar to those reported in recent outbreaks [6]–[9], [11]–[14], [16]–[18], [21], [30], indicating that although people are genetically diverse response to diseases is homogeneous across people in non-homogeneous populations. The majority of our patients was ≤45 years and had no pre-morbid condition. Unlike patients reported in the Reunion Island outbreak [6], where the patients' underlying medical conditions could have contributed to the observed morbidities, our patients were younger and healthier. Furthermore, none of our patients was co-infected with dengue, as confirmed by RT-PCR and dengue enzyme-linked immunoabsorbent assay (ELISA)-IgM and IgG [31]. Hence, our immunologic observations can be largely attributed to acute CHIKV infection itself.

In recent years, most of the studies on CHIKF have been addressed with the clinical description of the disease [4]–[9], [11], [17], [18], [21], the molecular nature of the virus [9], [10], [19] and diagnostics methods [8]–[10], [24], and the interactions of the virus with its mosquito vector, *Aedes* [1]–[3], [11]–[15]. Here, we describe for the first time the comprehensive systemic production of cytokines, chemokines, and growth factors during acute CHIKV infection which may light the path ahead in understanding the innate response to the infection. We first showed that a wide range of cytokines such as IFN-α, IL-5, IL-6, IL-7, IL-10, IL-15 were produced in response to CHIKV infection. IFN-α is a potent anti-viral cytokine and has been shown to strongly inhibit CHIKV *in vitro* [32]. The high levels of IFN-α that we detected provide a logical explanation for how the body rapidly brings CHIKV viremia under control [8], [11]. It has been shown that the main producers of IFN-α are plasmocytoid dendritic cells [33] and monocytes [34].

The profile of circulating cytokines revealed a predominance of Type 2 cytokines. Mainly IL-5, IL-6 and IL-10 levels were increased and those of IFN-γ or TNF-α were unchanged as compared to non-infected controls. This suggests that acute CHIKV infection tilts the cytokine profile to anti-inflammatory response, which would argue against the common understanding of CHIKV infection which does not really support the common description of the CHIKV infection as an inflammatory disease [8]. Alternatively, it is possible that an inflammatory response might occur earlier when the virus is actively replicating, and then gets down-regulated by a counter-anti-inflammatory response when the virus is being eliminated from circulation. High levels of anti-inflammatory IL-10 and the presence of high levels of chemokines IP-10 and MIG (ligands of CXCR3 associated with Th1-type reactions) [35], detected here would support this hypothesis. Further studies would be needed to clarify this issue. The Type 2 cytokines detected are also important mediators of B cell growth and maturation, and thus may allow the production of high levels of persisting anti-CHIKV IgG [5].

The detection of high levels of circulating IL-15 is of interest, since this cytokine has been shown to be a major stimulator of NK cells [36] and T cells [37]. Thus our data suggest that these lymphocytes population might be activated during acute infection and may also contribute to viral control during the acute phase of CHIKV infection. Detection of soluble IL-2R in the plasma suggests T cell activation since this molecule is secreted by activated T cells [38]. Experiments are planned to study the activation phenotype of T and NK cell subsets in acutely infected patients.

The detection of IL-7 and IL-15 is significantly interesting with regards to the immunopathology of CHIKF since CHIKV infection has been shown to induce rapidly developing and persisting arthralgia [6]. Here, 9 of the 10 patients manifested this pathology. IL-7 is known to have an important role in the development of rheumatoid arthritis [39], while IL-15 has been associated with the development of joint inflammation [40]. It has been proposed that expansion of a particular IL15-induced NK cell subsets was responsible for this phenomenon [41]. The role of IL-15 and NK cells in the development of CHIKV arthralgia would definitively be worth investigating. We did not detect TNF-α in the plasma of the patients with acute CHIKV infection. This is surprising since this cytokine has been detected repeatedly in the blood of patients suffering from other arthritides such as rheumatoid arthritis and is known to be involved in the pathogenesis of these entities [42]. Thus, it is possible that CHIKV-induced arthralgia does not depend on TNF-α. Alternatively, TNF-α might be produced only locally. Analysis of synovial fluid or joint tissue immunohistochemistry would be necessary to provide important information on the role of TNF-α and other mediators.

Chemokines are crucial mediators of innate and adaptive immunity against various viral infections [43]. IP-10, and MIG had increased plasma levels during CHIKV infection. These two chemokines signal through the same receptor CXCR3 and thus might activate and direct migration of this T cell subset [35]. IL-8 and Eotaxin levels were lower than those of naive controls. Defining the exact contributions of these different chemokines will require further studies.

We also tested the presence of growth factors in the plasma of CHIKV-infected patients. HGF, FGF-basic and VEGF were produced at high levels and may reflect a physiological response to tissue destruction resulting for the viral infection. Interestingly, EGF levels were lower than in healthy controls. The low levels of EGF might be due to the concomitant decrease of platelets observed in infected patients since previous studies have shown that plasma levels of EGF are associated with circulating platelets [44].

Although limited, we had access to sufficient patients to perform data analysis in relation to the severity of the disease (severe illness was defined as fever >38.5°C, or maximum pulse rate >100 beats/minute, or nadir platelet count <100×10^9^/L). Using this definition, we observed that higher disease severity was associated with increased plasma levels of IL-1β and IL-6 and a decreased level in RANTES (Figure 3b). IL-1β and IL-6, whose levels are already high in the CHIKV infected patients, are potent endogenous pyrogens [45]–[48]. Therefore, elevations of IL-1β and IL-6 might account for the high fever in the severe cases. The increase production of IL-1β might also mediate the development of abrupt and persistent arthralgia since this cytokine is involved in the immunopathogenesis of many arthritic pathologies such as rheumatoid arthritis [49]. On the contrary, T cell chemokine RANTES levels were significantly suppressed in severe CHIKF patients. Platelets are a major reservoir of RANTES in the peripheral circulation [50], and severe CHIKF was characterized by thrombocytopenia. Thus, as mentioned above for EGF, thrombocytopenia can also reduce levels of circulating RANTES. Low levels of RANTES correlate with disease severity and mortality in individuals with severe malaria, who were also correspondingly thrombocytopenic [51]. Interestingly, it was observed in other studies that RANTES levels were up-regulated in dengue [48], except for one single report from Cuba [52]. Since the symptoms of CHIKF mimic those of dengue fever, results obtained from this study strongly suggest that RANTES could be a potential biomarker that differentiates between these 2 clinically very similar diseases.

One limitation of this study is in the classification of disease severity as none of our patients developed neurologic or hemorrhagic complications previously reported in CHIKF patients. Nonetheless, our definition of severe illness would have included patients with sepsis, a serious form of infection commonly associated with a temperature of >38°C and heart rate of >90/minute [24]. Furthermore, we included thrombocytopenia of <100×10^9^/L as a criteria for severe CHIKF. Marked thrombocytopenia is a common feature of sepsis [25] and has been identified as a predictor of mortality [26], [27]. The degree of thrombocytopenia is a determinant of survival and once the platelet count decreases below 100×10^9^/L, mortality continues to increase, even though the risk of bleeding does not [28]. A wide spectrum of disease has been reported in CHIKF ranging from asymptomatic infections, to self-limiting febrile illness [8], to neurologic complications, and death [17]. The “severe illness” cohort in our study possibly represents a more severe form of self-limiting febrile illness, an intermediate group with higher levels of viremia (data not shown) and distinctly more severe clinical features (i.e. high temperature, tachycardia, and severe thrombocytopenia). Using this clinical phenotype, we have shown in this study that immune mediators are able to distinguish very mild disease from more severe forms of CHIKF disease at the acute stage. Follow-up studies will be required to determine if long-term sequelae are indeed different between non-severe and severe clinical presentations. Elucidating the association of disease severity with two cytokines and one chemokine can be useful in order to provide early identification and monitoring of patients with severe disease. Although this study is limited by the size of the outbreak, nevertheless, based on these observations, measurement of immune mediators could be helpful for the management of future outbreaks. This study strongly suggests these biomarkers be used for measuring disease severity and be tested in outbreaks in different populations and different strains. Once confirmed, they will be useful for follow-up studies, association studies, and prognosis for public health management. More importantly, these biomarkers can potentially lead to the development of modulators to reduce disease severity and to halt disease progression.

## Methods

### Patients and Clinical Samples

Ten patients who presented with acute CHIKF to the Communicable Disease Centre at Tan Tock Seng Hospital (CDC/TTSH), the national infectious disease referral centre in Singapore, during the outbreak period from January to February 2008, were included in this study. An acute case of CHIKF was defined as any case with clinical features consistent with CHIKF, and had CHIKV infection confirmed by either reverse transcription-polymerase chain reaction (RT-PCR) or virus isolation [53], [54]. The study was approved by the institution's domain-specific ethics review board (DSRB Reference No. B/08/026). Written consent was obtained from each patient and healthy control subject.

Plasma samples were obtained from patients during the acute phase of their illness. Data on demographic characteristics, pre-morbid conditions, clinical features, and routine hematological and biochemical laboratory test findings (i.e. full blood count, renal and liver function tests, C-reactive protein) were also collected. All symptomatic patients were isolated at CDC/TTSH until the febrile illness resolved and a negative CHIKV RT-PCR test was obtained. During the hospital stay, daily monitoring of body temperature, vital parameters, and blood counts were carried out. A patient was defined as having severe illness, if he had either a maximum temperature of more than 38.5°C, or a maximum pulse rate of more than 100 beats/minute, or a nadir platelet count of less than 100×10^9^/L. Laboratory results were expressed as mean±SD.

In addition, plasma or serum samples from 9 healthy volunteers (who did not have a febrile illness in the preceding week and were not epidemiologically-linked to the outbreak) were also included as controls in our study.

### Multiplex Microbead Immunoassay

Plasma samples collected as described above, were aliquoted and stored at −80°C until analyses were done. A multiplex biometric immunoassay, containing fluorescent dyed microspheres conjugated with a monoclonal antibody specific for a target protein, was used for cytokine measurement according to manufacturer's instructions (Biosource Human Cytokine 30-plex Assay, Invitrogen). The following groups of cytokines were: inflammatory (GM-CSF, IL-1beta, IL-1RA, IL-6, IL-8, TNF-alpha); Th1/Th2 (IFN-gamma, IL-2, IL-2R, IL-4, IL-5, IL-10); Cytokine II (IFN-alpha, IL-7, IL-12p40/p70, IL-13, IL-15, IL-17); Chemokines (Eotaxin, IP-10, MCP-1, MIG, MIP-1alpha, MIP-1beta, RANTES); and Growth Factors (EGF, HGF, FGF-basic, G-CSF, VEGF). Briefly, 25 ul of plasma samples were diluted in 1∶2 and incubated with antibody coupled beads for 2 h at RT °C. Complexes were washed twice with the use of a vacuum manifold before incubation with biotinylated detector antibody for 1 h at RT °C. Complexes were then washed twice followed by incubation with Streptavidin-phycoeryrhrin (RPE) for 30 min at RT °C. Complexes were washed thrice and incubated with wash buffer for another 3 min before detection in the Luminex 200™ instrument. Results were acquired by the IS 2.3 software and the standard curves were plotted through a five-parameter logistic curve setting.

### Algorithms for Data Analyses

To compare and analyze the expression profiles across the results, the raw cytokine values were normalized using z-score conversion based on the formula:
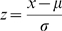
where 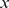 is the raw value to be converted, 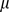 is the mean of the population and 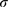 is the standard deviation (SD) of the population. The transformed value is denoted by 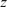 and exhibits positive value when the raw value is above mean and vice versa. Further, to examine the nuances and correlations masked in the full set of data, the 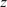 values are subjected to cluster analysis [55] to yield an ordered NumOfRows.x NumOfCols expression level matrix, *E_el_*. Hierarchical clustering is applied on the columns which represent the myriad of cytokine levels measurements (e.g.) based on McQuitty's or WPGMA method [56] where the distance between a pair of groups A and B is measured using the weighted arithmetic mean of all the pairwise distances between the data points in A and B. The rows which represent the suspected CHIKF patients and healthy individuals are left untouched. Seriation [57] is performed following the clustering approach to re-order the clustered data points using the minimum path length algorithm to minimize the sum of all the distances between adjacent columns.
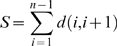
Euclidean distance is used in both the clustering and seriation phases to measure the difference or dissimilarity, *d*, between data points (x_1_, y_1_) and (x_2_, y_2_) given by the equation:
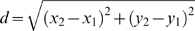



### Statistical Analyses

Comparisons between groups were calculated by Mann-Whitney rank sum. Further statistical analyses were done by Kruskal-Wallis test followed by Dunn's multiple comparison tests. *P* values of *<0.05* were considered to be statistically significant.

## Supporting Information

Figure S1 Complete profile of the levels (pg/ml) of cytokines, chemokines and growth factors determined by multiplex-bead arrays from blood samples collected from CHIKV-infected patients and healthy control subjects.
(0.33 MB EPS)

Figure S2 Box-and-whisker graphs of 14 immune mediators (cytokines and chemokines) determined from blood samples collected from healthy control subjects and CHIKV-infected patients.
(0.40 MB EPS)

Figure S3 Box-and-whisker graphs of 13 Chemokines and growth factors determined from blood samples collected from healthy control subjects and CHIKV-infected patients.
(0.40 MB EPS)
